# Pretreatment of Huaiqihuang extractum protects against cisplatin-induced nephrotoxicity

**DOI:** 10.1038/s41598-018-25610-6

**Published:** 2018-05-09

**Authors:** Yujiao Guo, Meng Wang, Jingyi Mou, Zhi Zhao, Juan Yang, Fengming Zhu, Guangchang Pei, Han Zhu, Yuxi Wang, Gang Xu, Rui Zeng, Ying Yao

**Affiliations:** 1 Department of Nephrology, Tongji Hospital, Tongji Medical College, Huazhong University of Science and Technology, Wuhan, China; 2 Department of Radiology, Tongji Hospital, Tongji Medical College, Huazhong University of Science and Technology, Wuhan, China; 3 Department of Pediatric, Tongji Hospital, Tongji Medical College, Huazhong University of Science and Technology, Wuhan, China

## Abstract

Cisplatin is a commonly used chemotherapeutic agent in the treatment of different types of malignant tumors, but nephrotoxicity limits its usage. Therefore, in this study, we aimed to determine the possible protective effect of Huaiqihuang (HQH) extractum, a kind of Chinese herbal complex that consists of *Trametes robiniophila* Murr., *Lycium barbarum* and *Polygonatum sibiricum*, against nephrotoxicity induced by cisplatin in mice. We found that pretreatment with HQH significantly attenuated the cisplatin-induced increase in blood urea nitrogen (BUN), interstitial congestion, acute renal tubular injury and tubular cell apoptosis and necroptosis. It was further shown that HQH administration reduced cisplatin-induced release and nuclear-cytoplasmic translocation of HMGB1 and inactivated its downstream signaling molecules, TLR4 and NFκB, in renal tubular cells; as a result, HQH repressed cisplatin-induced TNF-α production. As dexamethasone (Dex) exerts renoprotective effects in severe Acute kidney injury (AKI), we compared it with HQH and found that HQH showed similar renoprotective effects to dexamethasone via similar mechanisms. Considering the potential side effects of corticosteroids, reducing the effectiveness of treatment and shortening survival in solid tumor patients, we suggest administration of HQH as a potential adjuvant for cisplatin therapy in solid tumor patients to preserve renal function.

## Introduction

Cisplatin is a widely used anti-cancer agent that has been used in non-small cell lung, esophagus, bladder, endometrium, ovary and testicular cancers, as well as many other types of cancers^1,2^. However, its side effects, such as ototoxicity, renal toxicity and neurotoxicity, limit its clinical use^3–5^; among these effects, nephrotoxicity is the most concerning. Nephrotoxicity induced by cisplatin mainly occurs in renal tubular proximal tubular epithelial cells and is characterized by tubular cell necrosis, oxidative damage, inflammatory injury and acute renal failure^6–8^, which is related to high morbidity and mortality^9^. As the mechanisms implicated in cisplatin-induced AKI are still unclear, an effective clinical agent for preventing cisplatin-induced AKI is needed. Recently, dexamethasone (Dex) has been used as a potent therapeutic agent for acute kidney injury induced by cardiac surgery^10,11^, multiple myeloma^12,13^ and drug-induced nephrotoxicity^14,15^. However, the shortened survival of solid tumor patients due to corticosteroids restricts its application^16^.

Huaiqihuang (HQH) extractum is a Chinese herbal complex that consists of *Trametes robiniophila* Murr., *Lycium barbarum* and *Polygonatum sibiricum*, all of which have been used intensively in China for more than 1, 000 years. Among them, *Trametes robiniophila* Murr. is one of the important anti-tumor herbs and is widely used in the adjuvant therapy of primary liver cancer, breast cancer, stomach cancer, colon cancer and other cancers^17–20^. HQH extractum has been used in the treatment of nephrotic syndrome, bronchial asthma and recurrent upper respiratory tract infection in children. An *in vitro* study showed that HQH extractum protects against podocyte injuries by regulating the p-ERK/CHOP signaling pathway to promote podocyte proliferation and suppress podocyte apoptosis^21^. Liu H *et al*. reported that HQH extractum could increase podocyte nephrin expression, inhibit the NF-κB signaling pathway and suppress glomerular and tubular apoptosis in rats with adriamycin-induced nephropathy^22^. All these studies suggest that HQH extractum is a potent nephroprotective agent; however, little is known about the protective effect of HQH in AKI.

In this study, the renoprotective effects of HQH against cisplatin-induced AKI was surveyed in mice and we found that pretreatment with HQH demonstrated renoprotective effects similar to those of Dex via similar mechanisms to relieve cisplatin-induced renal injury. Furthermore, it was shown that HQH did not impair cisplatin-mediated killing of HeLa tumor cells *in vitro*. Taken together, these results suggest that HQH is a potent renoprotective agent for preventing the renal toxicity caused by cisplatin without affecting the anti-tumor effects of cisplatin.

## Results

### Effects of Huaiqihuang (HQH) Extractum on Renal Damage in Cisplatin-Treated Mice

**Figure 1:**
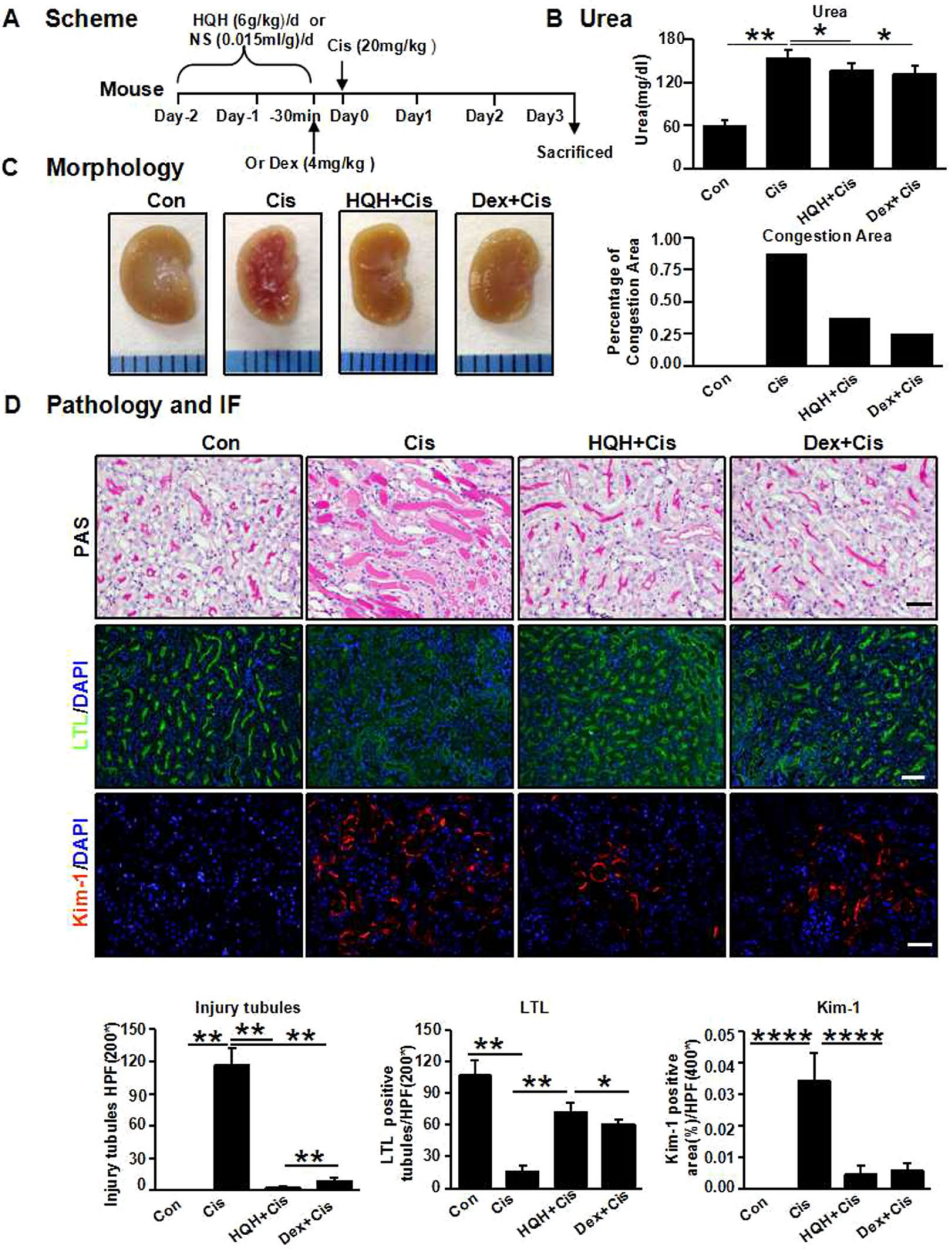
HuaiQihuang extractum (HQH) or Dexamethasone (Dex) pretreatment attenuated acute renal injury induced by cisplatin (Cis). (**A**) Scheme: The group of HQH + Cis received 6 g/kg HQH for two consecutive days before intraperitoneal injection with 20 mg/kg cisplatin and the group of Dex + Cis intraperitoneally injected with 4 mg/kg Dex 30 minutes before intraperitoneal injection with cisplatin. (**B**) Serum urea was measured at day 3 after cisplatin injection (*P < 0.05, **P < 0.01, n = 5/group). (**C**) Representative medullary congestion after Cisplatin injury with or without HQH and Dex pretreatment. Graphs indicate congestion area. (**D**) Representative photomicrographs and graphs of Cisplatin induced kidney injury, with or without HQH and Dex pretreatment. Injured tubules was showed by PAS staining, Bar = 50 μm. LTL (lotus tetragonolobus lectin) identifying proximal tubules (green), Bar = 100 μm. Kim-1 expression (red) identifying tubular injury, Bar = 50 μm. Statistics analyzed using the Mann-Whitney U test. **P < 0.01, ****P < 0.0001, n = 5/group. Values are means ± SEM.

The experimental design of the renoprotective effect of HQH on cisplatin-induced renal injury in C57BL 6 male mice is summarized in Fig. 1A. Urea levels were significantly increased in the cisplatin-treated group, but this effect was significantly reversed by HQH and Dex pretreatment (Fig. 1B). An obvious medullary congestion zone was seen in cisplatin-treated kidneys when the mice were sacrificed on day 3, which was significantly attenuated after HQH pretreatment (Fig. 1C).

The morphological changes in the kidneys are shown in Fig. 1D. Cisplatin-induced cast formation, tubular dilation and brush border loss were significantly alleviated by HQH pretreatment (Fig. 1D). The HQH group showed less tubular cast than the Dex group.

To assess the injury of proximal renal tubule cells (TECs), we detected the brush border with immunofluorescence staining of lotus tetragonolobus lectin (LTL) and kidney injury molecule 1 (Kim-1). As shown in Fig. 1D, the loss of LTL-positive tubules in the cisplatin-treated kidneys was restored by HQH pretreatment. Compared to the Dex group, the HQH group had more LTL-positive tubules. Compared to its expression in cisplatin-treated control mice, Kim-1 was less expressed in the HQH group, similar to the Dex group, (Fig. 1D). These data suggested that HQH pretreatment alleviated cisplatin-induced AKI better than the dexamethasone.

### Effects of Huaiqihuang (HQH) Extractum on Proliferation, Apoptosis and Necroptosis of TECs in Cisplatin-Treated Mice

**Figure 2:**
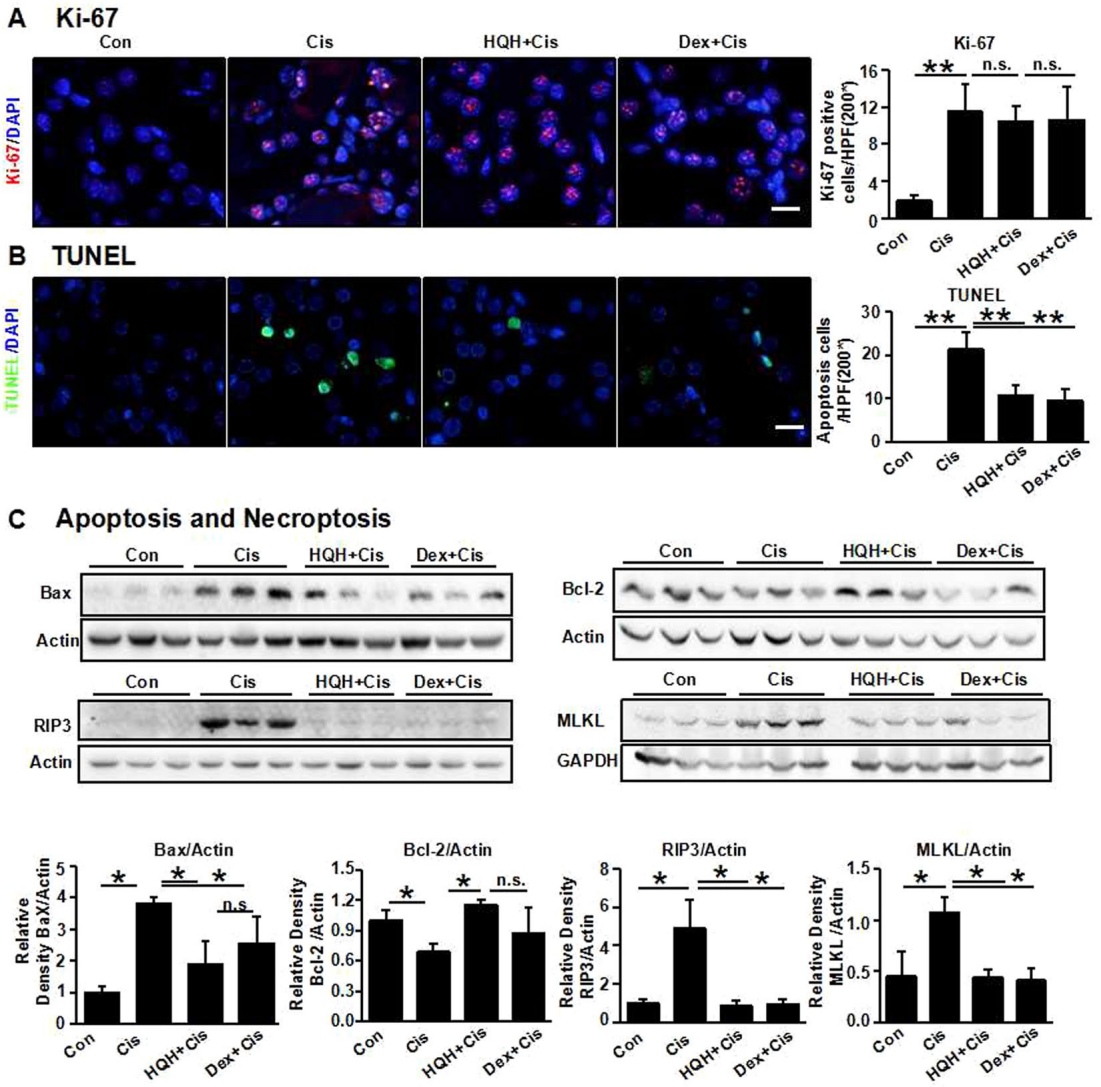
HQH pretreatment protected mice from cisplatin induced tubular apoptosis and necroptosis, but not improved renal tubular epithelial cell proliferation. (**A**) Representative photomicrographs and graphs of immunofluorescence staining for Ki-67. Bar = 50 μm. (**P < 0.01 versus control, n = 8/group). Ki-67 was stained in red and nucleus was counterstained in blue by DAPI. (**B**) Representative photomicrographs and graphs of TUNEL (green) assay for analysis of tubular apoptosis (**P < 0.01, n = 5/group). Bar = 50 μm. (**C**) Representative Western blot and graphs for pro-apoptotic proteins Bax (**C** and **D**, *P < 0.05, n = 3/group), anti-apoptotic protein Bcl-2 (**E** and **F**, *P < 0.05, n = 3/group) and the key element of necroptosis RIP3 (*P < 0.05, n = 3/group) and MLKL (*P < 0.05, n = 3/group). Each group of blots is the same exposure of a gel, each group of results repeated more than three times.

Previous studies have shown that cisplatin-induced AKI is accompanied by proliferation of renal TECs^23–25^. We found that cisplatin induced a significant increase in Ki67-positive nuclei in TECs, which was not further increased in the HQH- and Dex-pretreated group (Fig. 2A), suggesting that HQH pretreatment-induced renoprotection does not occur through the increased renal regeneration capacity related to TEC proliferation. Apoptosis in TECs was assessed using the terminal deoxynucleotidyl transferase–mediated dUTP nick-end labeling (TUNEL) assay (Fig. 2B). TUNEL-positive cells were almost undetectable in the control kidneys, while the number of the TUNEL-positive cells was significantly increased after cisplatin administration. Pretreatment of both HQH and Dex reduced the cisplatin-induced apoptosis; however, there was no difference between the HQH-pretreated group and the Dex-pretreated group. We further examined the expression of the pro-apoptotic protein Bax and the anti-apoptotic protein Bcl-2, which showed that HQH pretreatment attenuated cisplatin-induced Bax expression (Fig. 2C) and enhanced the expression of anti-apoptotic Bcl-2. This result suggests that HQH pretreatment protected the kidneys against cisplatin-induced apoptosis. Tubular necroptosis is another characteristic of kidney tissues in cisplatin-treated mice and is characterized by high levels of receptor interacting-protein 3(RIP3) and mixed-lineage kinase domain-like protein (MLKL) in TECs^26^. To assess this necroptosis, we observed the expression of RIP3 and MLKL by western blotting (Fig. 2C). We found that both HQH and Dex pretreatment attenuated kidney necroptosis induced by cisplatin, suggesting that HQH treatment protected the kidneys against cisplatin-induced necroptosis.

### Effects of Huaiqihuang (HQH) Extractum on inflammation of kidneys in Cisplatin-Treated Mice

**Figure 3:**
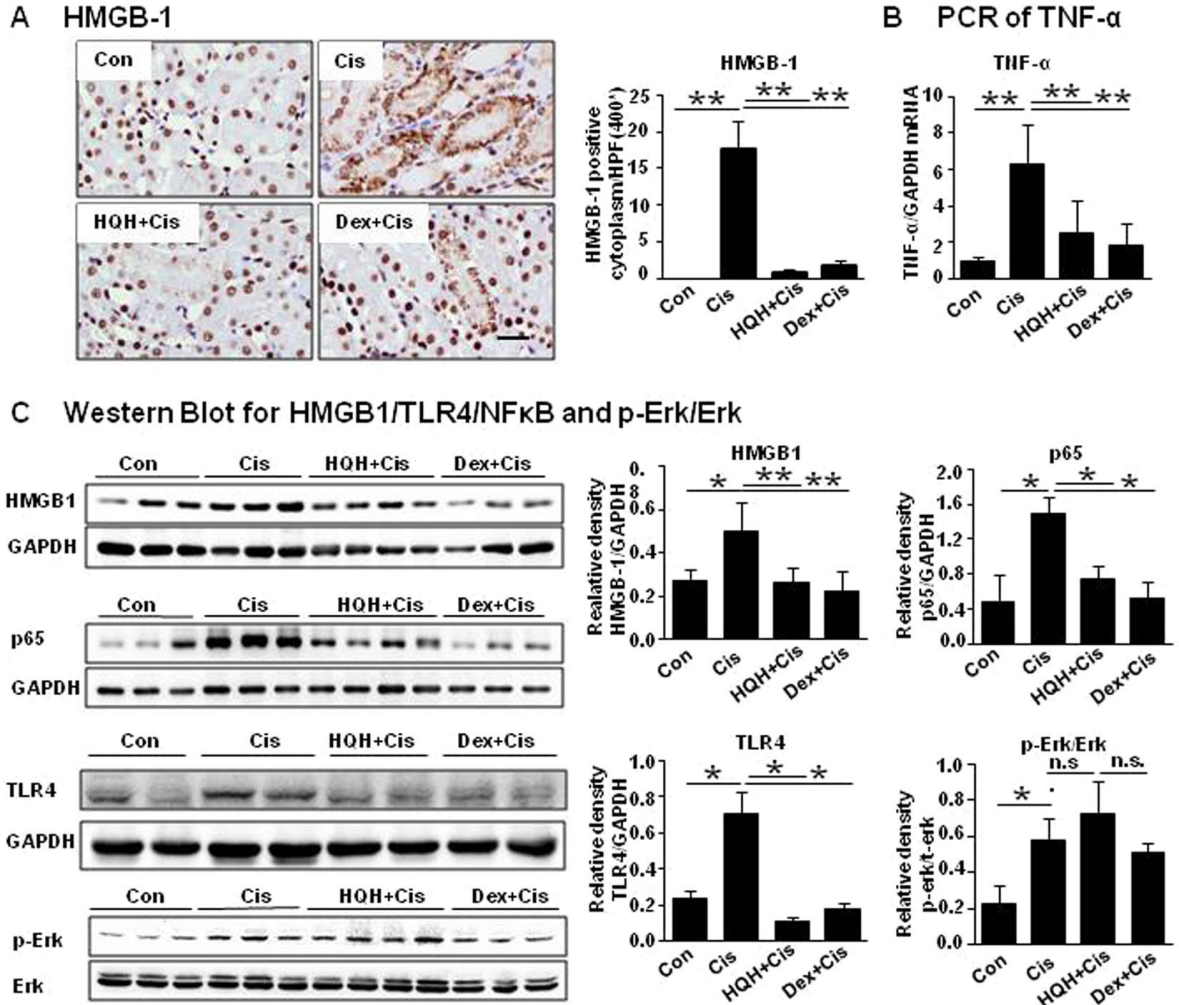
HQH protected the kidney through inhibition of HMGB1/TLR4/NF-κB/TNF-α pathway. (**A**) Representative photomicrographs and graphs for the expression of HMGB1 by immunohistochemical staining (**P < 0.01, n = 5/group). Bar = 50 μm. (**B**) Real-time PCR analyses of TNF-α (**P < 0.01, n = 3 ~ 7/group). (**C**) Representative Western blot and graphs for HMGB-1 in cytoplasmprotein (**P < 0.01, *P < 0.05, n = 3 ~ 4/group) and p65 in nucleoprotein (*P < 0.05, n = 3 ~ 4/group), and TLR4 (*P < 0.05, n = 4/group), p-Er and Erk (*P < 0.05, n = 3/group) in total protein. Each group of blots is the same exposure of a gel, each group of results repeated more than three times.

Previous study confirmed that cisplatin-induced acute renal injury is related to the release of pro-inflammatory cytokines, including high mobility group box-1 protein (HMGB1) and tumor necrosis factor-α (TNF-α)^27^. As shown in Fig. 3A, cisplatin injection resulted in obvious release of HMGB1 into the cytoplasm in TECs, which was restored by HQH and Dex pretreatment (Fig. 3A). We further used RT-PCR to detect the expression of TNF-h (Fig. 3B). We found that both HQH and Dex pretreatment reduced the expression of TNF-N induced by cisplatin. These results were further confirmed by Western blotting, as shown in Fig. 3C. NFκB and TNF-N are the downstream signaling pathways of HMGB1 and TLR4^28–30^. We found that HMGB1 in the cytoplasm, NF-κB P56 in the nucleus and TLR4 in total protein, were highly induced after cisplatin administration and these effects were reversed by HQH and Dex pretreatment (Fig. 3C). We also assessed the MAPK signaling pathway; however, the expression of p-Erk/Erk was increased in all three groups and there was no significant difference between them (Fig. 3C). This result suggests that HQH pretreatment protected the kidneys against cisplatin-induced inflammation through the HMGB1/TLR4/NFκB pathway.

### HQH Extractum does not Bind to Cisplatin

**Figure 4:**
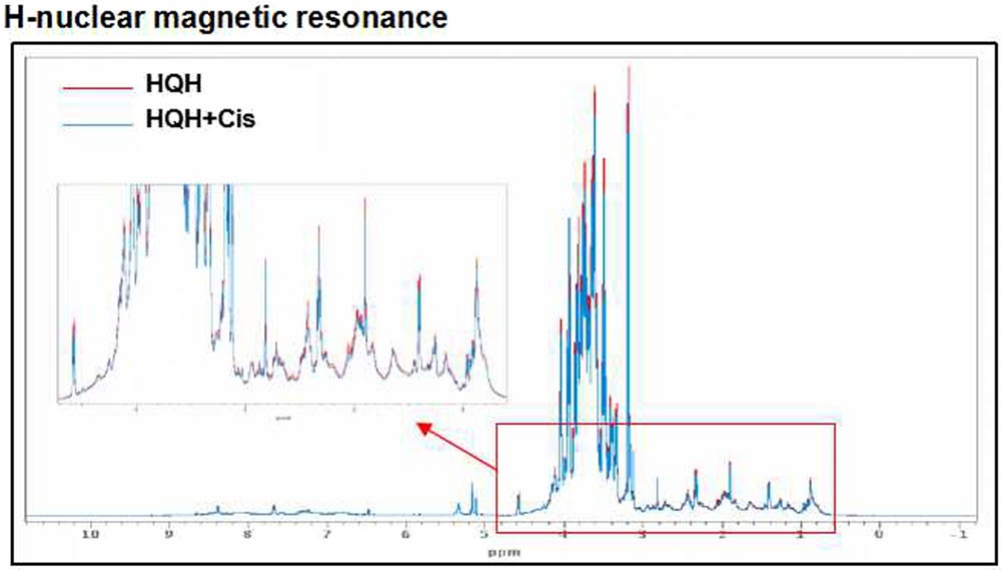
HQH does not bind with cisplatin. (**A**) The nuclear magnetic resonance showed that HQH and Cis does not interact with each other. The red line indicates the hydrogen spectra of HQH. The blue line indicates the hydrogen spectra of which Cis into HQH. These two lines substantially overlapped, suggesting that HQH does not interact with Cis. (nuclear magnetic resonance spectrometer, agilent 600 DD2).

To observe the change in HQH after the addition of cisplatin, we applied nuclear magnetic resonance to detect the change in the one-dimensional proton spectrum. If cisplatin binds to HQH, it will cause chemical shifts, which will cause a change in the proton spectrum. However, we found that these two lines substantially overlapped (the red line indicates the hydrogen spectra of HQH and the blue line indicates the hydrogen spectra of Cis added to HQH) (Fig. 4), suggesting that HQH and cisplatin do not undergo chemical or physical reactions and that neither agent produces chelates or degradation. In other words, HQH does not react with cisplatin to prevent cisplatin from entering the cell.

### HQH Extractum does not Impair Cisplatin-Mediated Killing of HeLa Tumor Cells *In Vitro*

**Figure 5:**
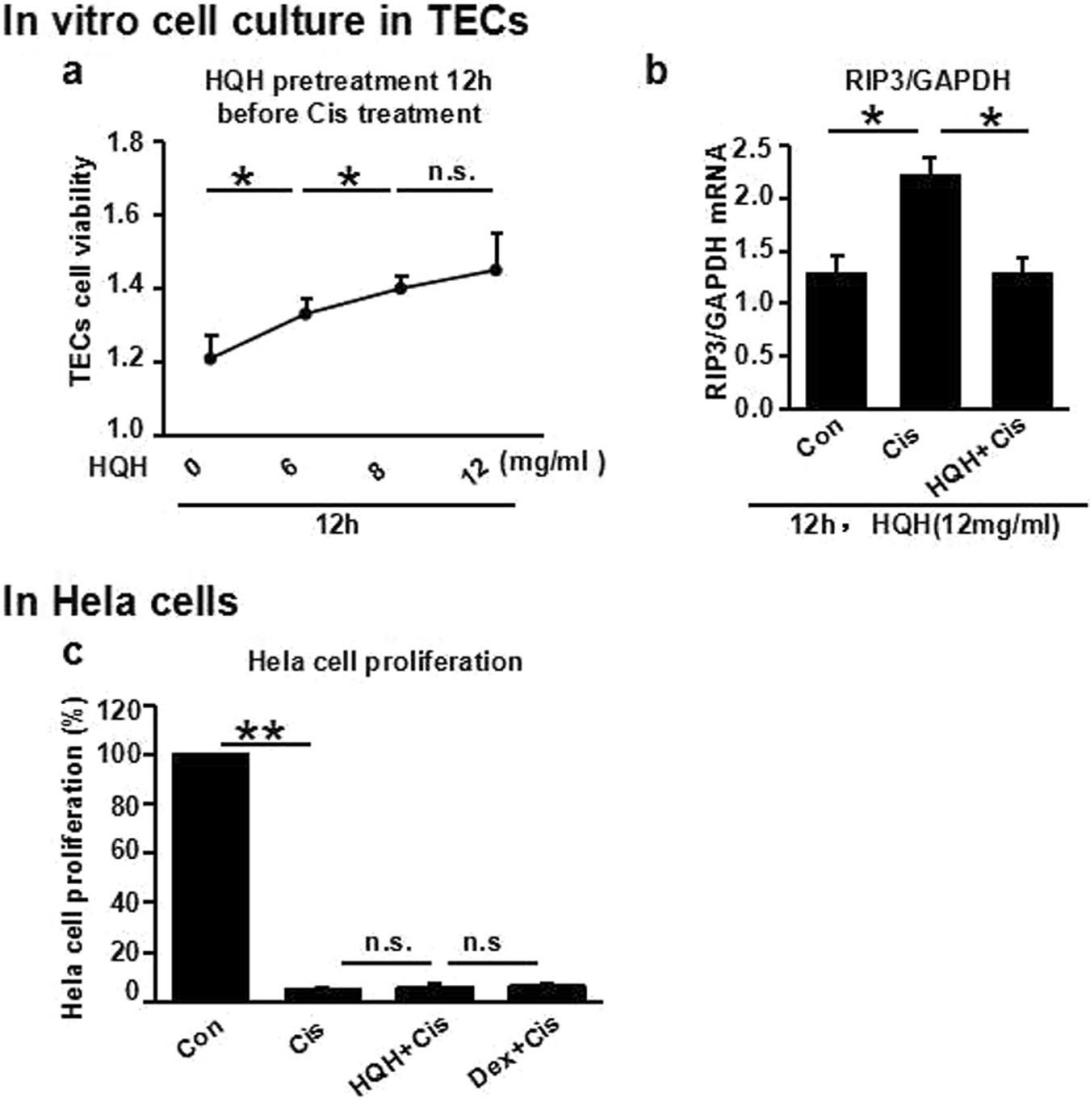
HQH reduced the damage of renal tubular epithelial cells induced by cisplatin, but did not affect the antitumor activity of cisplatin in Hela cells. (**a**) CCK 8 experiment in renal proximal tubules after HQH (0/6/12/18 mg/ml) pretreatment 12 h before cisplatin (75 μM) treatment 12 h (*P < 0.05, n = 5/group). (**b**) RIP3 expression are decreased in renal proximal tubules after HQH (12 mg/ml) pretreatment 12 h before cisplatin (75 μM) treatment 12 h (*P < 0.05, n = 5/group). (**c**) CCK8 cell proliferation assay for Hela cell. Con: Without stimulus; Cis: 30 mM; HQH: 10 mg/ml; Dex: 50 μM; (**P < 0.01, n = 5/group).

To verify the protective effect of HQH on the kidney at the cellular level, we performed a CCK 8 experiment in cisplatin-treated renal tubular epithelial cells after HQH pretreatment, which showed that HQH pretreatment increased the viability of TECs in a dose-dependent manner from 0 mg/mL to 12 mg/mL (Fig. 5a). Therefore, we pretreated TECs with HQH at a concentration of 12 mg/mL and showed that, at such a dose, HQH pretreatment significantly inhibited cisplatin-induced necroptosis *in vitro* (Fig. 5b). Thus, we used 12 mg/mL HQH for the following *in vitro* experiments in tumor cells (HeLa cells). We found that HQH does not inhibit the anti-tumor activity of cisplatin in HeLa cells (Fig. 5c), which suggests that HQH pretreatment protects against cisplatin-induced renal toxicity without affecting the anti-tumor effect of cisplatin.

### HQH Extractum Pretreatment Attenuates AKI In Two Other Models

**Figure 6:**
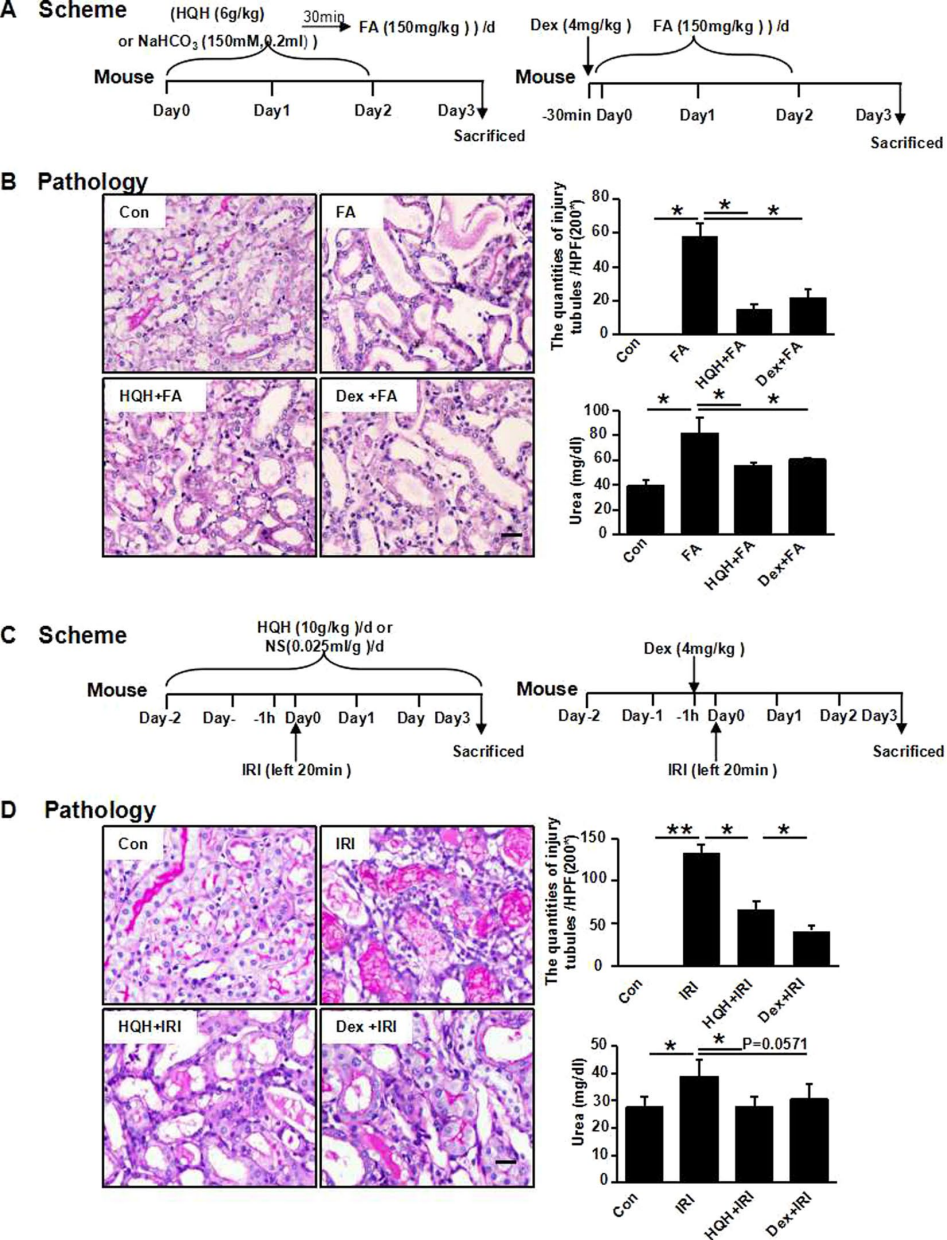
HQH and Dex pretreatment attenuate renal injuries induced by folic acid (FA) or unilateral kidney ischemia reperfusion (IRI). (**A**) Scheme: Male C57BL/6 mice with body weight about 25 g underwent intraperitoneal injection with NaHCO3(150 mM) or 150 mg/kg folic acid (n = 4/group). Mice received gavage of 6 g/kg HQH extractum every day or one dose of Dex with 4 mg/kg before intraperitoneal injection with 150 mg/kg folic acid. (**B**) Representative photomicrographs and graphs of kidney injury outcomes induced by folic acid (*P < 0.05, n = 4/group). Bar = 100 μm. Serum urea was measured at day 3 (*P < 0.05, n = 4/group). (**C**) Scheme: Male C57BL/6 mice underwent left kidney Ischemia Reperfusion Injury (IRI) (ischemia 20 min, n = 5/group). The group of HQH + IRI mice received 10 g/kg HQH extractum gavage two consecutive days before IRI surgery on left kidney and sacrificed three days later (ischemia 20 min, n = 5/group). The group of Dex + IRI mice intraperitoneal injected with 4 mg/kg Dex one hour before left kidney IRI surgery (ischemia 20 min, n = 5/group). (**D**) Representative photomicrographs and graphs of the kidney outcomes of the IRI models (*P < 0.05,**P < 0.01, n = 4 ~ 5/group). Bar = 50 μm. Serum urea was measured at day 3 after IRI (*P < 0.05, n = 4 ~ 5/group).

To verify that Huaiqihuang also provides protection in other nephrotoxic models, we established a folic acid (FA)-induced AKI model and an ischemia-reperfusion (IRI)-induced AKI model. The experimental design of the renoprotective effect of HQH on FA- or IRI-induced renal injury is summarized in Fig. 6A and C, respectively. FA-induced (Fig. 6C) and IRI-induced (Fig. 6D) cast formation, tubular dilation and brush border loss were significantly alleviated with HQH and Dex pretreatment. Urea levels were significantly increased by FA and IRI treatment, which were restored by HQH and Dex pretreatment (Fig. 6C and D). There was no difference between the HQH- and Dex-pretreated groups, suggesting that HQH pretreatment attenuated acute renal tubular injury and improved kidney function in various models of AKI in mice.

## Discussion

This study identified the renoprotective properties of HQH extractum on cisplatin-induced acute kidney injury. HQH does not bind to cisplatin directly *in vivo*. It ameliorated cisplatin-induced TECs apoptosis and necrosis and preserved renal function by inhibition of the HMGB1/TLR4/NFκB/TNF-α pathway. We found that HQH pretreatment did not inhibit the anti-tumor activity of cisplatin and it demonstrated similar renoprotective effects in various models of AKI, similar to Dex. In considering the reports that corticosteroids retard the treatment effectiveness of solid tumors and shorten survival to a certain extent^16,31,32^, the present findings opened a new market for the prevention of kidney injury in patients with solid tumors.

Cisplatin-induced nephrotoxicity involves a complex multifactorial process. First, cisplatin has a direct toxic effect on the renal tubular cells and renal blood vessels, resulting in a decline in renal blood flow and glomerular filtration rate. Second, cisplatin induces renal interstitial inflammation and chronic interstitial fibrosis, leading to irreversible renal damage. Additionally, cisplatin activates a variety of cellular pathways, cytokines and signaling molecules in TECs, which causes renal inflammation, apoptosis and necrosis^9,33–35^.

Huaiqihuang (HQH) extractum is a Chinese herbal complex composed of *Trametes robiniophila* Murr. (Huaier), *Lycium barbarum* (Gouqi) and *Polygonatum sibiricum* (Huangjing). It has been identified that Huaiqihuang may protect against proteinuria by preventing MPC5 podocyte damage via targeting the p-ERK/CHOP pathway^21^ and enhancing Nephrin expression and the necrosis factor κg signaling pathway^22^. HQH not only activates macrophages, neutrophils, natural killer cells but also promotes T lymphocyte division, proliferation, maturation and differentiation^36^. Based on the immunoregulatory function of HQH extractum and the possible direct protective effect on injured kidneys, mice were pretreated with HQH before the injection of cisplatin. As a result, the number of renal tubular epithelial cell injuries, swelling, apoptosis and necrosis was decreased and after pretreatment with HQH, the renal tubular lesion was alleviated, which was significantly lower than that of the cisplatin-treated control group (P < 0.05). Such protective effects were further identified in FA- and IRI-induced AKI models (Fig. 6), suggesting that HQH is a broad-spectrum nephroprotective agent for AKI.

Glucocorticoids have been largely used in the clinical treatment of acute renal injury with a significant protective effect^37–39^, but the side effects of glucocorticoids for cancer patients, such as hair loss, endocrine disorders, weight gain and osteoporosis, make them unsuitable for long-term use. The more serious concern is that corticosteroids even inhibit the effectiveness of cancer treatment and shorten survival of solid tumor patients^16,31,32^. However, the key component of HQH, *Trametes robiniophila* Murr. (Huaier), has been identified as an effective chemotherapeutic agent for certain tumors, ‘with safe and broad anti-cancer spectrum’^40–44^. In the present study, we found that pretreatment with HQH and dexamethasone showed a similar renoprotective effect for cisplatin-induced acute kidney injury treatment. Thus, HQH can eventually be used in clinical prevention or treatment of cisplatin chemotherapy-induced kidney injury.

In summary, the current study indicated that HQH extractum protects the kidneys from cisplatin-induced injuries by inhibiting the HMGB1/TLR4/NFκB/TNF-α pathway in renal tubular cells. It shows potent therapeutic activity superior to that of dexamethasone for AKI in cancer patients. HQH extractum might be a potential adjuvant for clinical cisplatin therapy.

## Materials and Methods

### Cisplatin-Induced Acute Kidney Injury Model

Male C57BL/6 mice (7 weeks old, 20–23 g) were purchased from the Hubei Experimental Animal Research Center. All mice were housed in the experimental animal center at the Tongji Medical College, Huazhong University of Science and Technology with a 12/12-h light/dark cycle. After a minimum of 1 week of acclimatization, the mice (n = 5/group) were randomly divided into four groups. Two groups of mice were gavage pretreated daily with normal saline (NS) for 3 days. The third group was gavage pretreated daily with HQH (6 g/kg; Qidong GaiTianLi Pharmaceutical Co.; dissolved by NS, 0.4 g/ml) for 3 days. A single nephrotoxic dose of cisplatin (20 mg/kg; Nanjing Pharmaceutical Co.) was administered via intraperitoneal injection (ip) to the HQH-pretreated group and one NS-pretreated group on the third day 30 minutes before the pretreatment. A single dose of NS (0.015 ml/g) was administered ip to one NS-pretreated group as a control. The last group was administered ip a single dose of dexamethasone (Dex) (4 mg/kg; MaAnShan Fengyuan Pharmaceutical Co.) 30 minutes before cisplatin (20 mg/kg) ip. The mice were sacrificed on Day 3. All studies were approved by the Animal Care and Use Committee (ACUC) in Tongji Hospital and conducted in accordance with NIH guidelines.

### FA-Induced Acute Kidney Injury Model

Male C57BL/6 mice (7 weeks old, 20–23 g) were purchased from the Hubei Experimental Animal Research Center. All mice were housed in the experimental animal center at the Tongji Medical College, Huazhong University of Science and Technology with a 12/12-h light/dark cycle. After a minimum of 1 week of acclimatization, the mice (n = 4/group) were randomly divided into four groups. Two groups of mice were gavage pretreated daily with normal saline (NS) for 3 days. The third group was gavage pretreated with HQH (6 g/kg; Qidong GaiTianLi Pharmaceutical Co.; dissolved by NS, 0.4 g/ml) daily for 3 days. A single nephrotoxic dose of FA (150 mg/kg; SIGMA; dissolved by 150 mM NaHCO_3_) was administered ip to the HQH-pretreated group and one NS-pretreated group per day 30 minutes after the gavage. A single dose of NaHCO_3_ (150 mM, 0.2 ml) was administered ip to one NS-pretreated group as a control. The last group was administered ip a single dose of Dex (4 mg/kg; MaAnShan Fengyuan Pharmaceutical Co.) 30 minutes before FA (150 mg/kg) ip. The mice were sacrificed on Day 3. All studies were approved by the Animal Care and Use Committee (ACUC) in Tongji Hospital and conducted in accordance with NIH guidelines.

### IRI-Induced Acute Kidney Injury Model

Male C57BL/6 mice (7 weeks old, 20–23 g) were purchased from the Hubei Experimental Animal Research Center. All mice were housed in the experimental animal center at the Tongji Medical College, Huazhong University of Science and Technology with a 12/12-h light/dark cycle. After a minimum of 1 week of acclimatization, the mice (n = 5/group) were randomly divided into four groups. Two groups of mice were gavage treated daily with normal saline (NS) for 5 days. The third group was gavage treated with HQH (10 g/kg; Qidong GaiTianLi Pharmaceutical Co.; dissolved by NS, 0.4 g/ml) daily for 5 days. Left renal ischemia-reperfusion surgery was performed on mice in the HQH-pretreated group and one NS-pretreated group one hour after the gavage on Day 0. No surgery was performed on the mice in one NS-treated group, used as controls. The last group was administered ip of a single dose of Dex (4 mg/kg; MaAnShan Fengyuan Pharmaceutical Co.) one hour before the left renal ischemia-reperfusion surgery. The mice were sacrificed on Day 3. All studies were approved by the Animal Care and Use Committee (ACUC) in Tongji Hospital and conducted in accordance with NIH guidelines.

### Serum Metabolites

Commercial kits were used to measure urea (Bioassay Systems, Hayward, CA).

### Histologic, immunohistochemical and immunofluorescence staining

Paraffin-embedded renal sections (3 μm) were subjected to periodic acid-Schiff (PAS) staining to evaluate tubular injury. The tubule injuries include cast formation, tubular dilation and brush border loss, which was analyzed by the quantities of injury tubules in >10 random high-power fields (HPF). LTL (Lotus Tetragonolobus Lectins) carrying green fluorescence could be seen on the tubular brush border with specific coloring at the proximal end. However, when external factors or internal immune factors lead to renal tubular injury the brush edge is removed and there is no fluorescence expression. Therefore, LTL low coloring can be used as an indicator of renal tubular injury. For immunohistochemical (IHC) analysis, renal sections (3 μm) were incubated with 3% H_2_O_2_ for 20 min and nonspecific proteins were blocked with 10% goat serum for 30 min at room temperature. The slides were incubated with primary antibodies against either Kim-1 (1:1000, R & D), Ki-57 (1:200, Abcam), or HMGB-1 (1:200, Abcam) at 4 °C overnight. The slides were then incubated with the appropriate secondary antibody (HRP-conjugated secondary antibody for IHC and fluorescent-labeled secondary antibody for IF).

### Western blot analysis

Renal tissues were homogenized in RIPA lysis buffer containing two kinds of protease inhibitors: cocktail (50×) and phenylmethanesulfonyl fluoride (PMSF, 100×). The extraction of nucleoprotein and cytoplasm protein was performed according to the manufacturer’s instructions (KeyGEN BioTECH). Total protein concentrations were determined using a BCA assay kit according to the manufacturer’s instructions. The kidney tissue total protein was separated by SDS-PAGE and the separated proteins were transferred to PVDF membranes. The membranes were blocked with 5% nonfat milk in TBS with 0.1% Tween-20 (TBST) for 1 h at room temperature and then probed with antibodies against either Bax (1:1000, Abclonal), Bcl-2 (1:1000, Abclonal), RIP3 (1:200, BioVision), MLKL (1:1000, Abgent), TLR-4 (1:500, Abgent), β-actin (1:2000, Google Bio.), p-Er/Erk or GAPDH (1:2000, Google Bio.) at 4 °C overnight and with p65 (1:1000, Rui Ying Bio.) for nucleoprotein and HMGB-1 (1:1000, Abcam) for cytoplasmic proteins. After being washed with TBST, the blots were incubated with an HRP-conjugated anti-IgG and the target bands were visualized with ECL plus reagents according to the manufacturer’s instructions. The intensities of the target bands were analyzed by densitometry and were normalized to GAPDH or β-actin using Quantity One software (BioRad, CA, USA); prior to analysis, the intensities of the target bands were normalized to the corresponding values of the control group.

### Real-time quantitative RT-PCR

Total RNA extraction and reverse transcription were conducted using a GoScript reverse transcription system (Promega, USA). PCR enzymes and master mixes (Thermo Scientific, USA) were used for real-time PCR with primers specific to mouse GAPDH and TNF-α. The sequences for all primers are shown in Table 1. Relative expression levels were normalized to GAPDH and calculated using the 2^−ΔΔCt^ method.

**Table 1:** Primer sequences.

	Forward	Reverse
Mouse GAPDH	5′-TCAACGATTTGGTCGTATT-3′	5′-CTGTGGTCATGAGTCCTTCC-3′
Mouse TNF-α	5′-CCCTCACACTCAGATCATCTTCT-3′	5′-GCTACGACGTGGGCTACAG-3′
Mouse RIP3	5′-CAGTGGGACTTCGTGTCCG-3′	5′-CAAGCTGTGTAGGTAGCACATC-3′

### Nuclear Magnetic Resonance (NMR)

First, 450 μl cisplatin (1 mg/ml) was taken and then 50 μl D_2_O was added; the samples were thoroughly mixed and placed in NMR tubes. One-dimensional ^1^H NMR spectra were obtained. Then, 500 μl H_2_O (containing 10% D_2_O) was added to 152.9 mg HQH and thoroughly mixed and after centrifugation, the supernatant was placed in NMR tubes. One-dimensional ^1^H NMR spectra were obtained. Next, 450 μl cisplatin with 50 μl D_2_O was added to 156.5 mg HQH and thoroughly mixed and after centrifugation, the supernatant was placed in NMR tubes. One-dimensional ^1^H NMR spectra were obtained.

### *In vitro* cell culture in TECs

Male C57BL/6 mice (4 weeks old, 12–15 g) were sacrificed and kidneys were removed; then, tubular epithelial cells were isolated and cultured in DMEM/F12 (Gibco) with 10% FBS medium at 37 °C in 5% CO_2_. Tubular epithelial cells were split into 96-well plates with HQH (0/6/12/18 mg/ml) when the cells were confluent. Then, a CCK8 experiment in renal proximal tubules was performed after HQH (0/6/12/18 mg/ml) pretreatment 12 h before cisplatin (75 μM) treatment for 12 h. The CCK8 experiment was performed according to the manufacturer’s instructions.

### *In vitro* cell culture in HeLa cells

HeLa cells were cultured in DMEM-H (Gibco) with 10% FBS medium at 37 °C in 5% CO_2_. The HeLa cells were split into 96-well plates with DMEM-H medium (with 1% FBS), 30 mM cisplatin, 10 mg/ml HQH + 30 mM cisplatin, or 50 μM dexamethasone + 30 mM cisplatin when the cells were confluent; then, a CCK8 cell proliferation assay for HeLa cells was performed. The CCK8 experiment was performed according to the manufacturer’s instructions.

### Statistical analysis

All statistics were analyzed using the Mann-Whitney U test; *P < 0.05, **P < 0.01, ***P < 0.001, ****P < 0.0001. Values are the means ± SEM. *P < 0.05 was considered to indicate a significant difference.

## Electronic supplementary material

Supplementary Information
