# Supplementary material for: Pretreatment of Huaiqihuang extractum protects against cisplatin-induced nephrotoxicity
Source: Sci Rep. 2018 May 9;8:7333. doi: 10.1038/s41598-018-25610-6 (PMC5943312; doi:10.1038/s41598-018-25610-6)

Pretreatment of Huaiqihuang extractum protects against cisplatin-induced nephrotoxicity

Yujiao Guo[1,2], Meng Wang [1], Jingyi Mou[3], Zhi Zhao[1], Juan Yang[1], Fengming Zhu [1],Guangchang Pei[1], Han Zhu[1], Yuxi Wang[1], Gang Xu[1], Rui Zeng[1,*], Ying Yao[1,*]

[1]Department of Nephrology, Tongji Hospital, Tongji Medical College, Huazhong University of Science and Technology

[2]Department of Radiology, Tongji Hospital, Tongji Medical College, Huazhong University of Science and Technology

[3]Department of Pediatric, Tongji Hospital, Tongji Medical College, Huazhong University of Science and Technology

*They contributed equally to corresponding author, 1095 Jiefang Ave, Wuhan, Hubei, China (postcode, 430030). (YY) E-mail:yaoyingkk@126.com; (RZ)E-mail:zengrui@tjh.tjmu.edu.cn

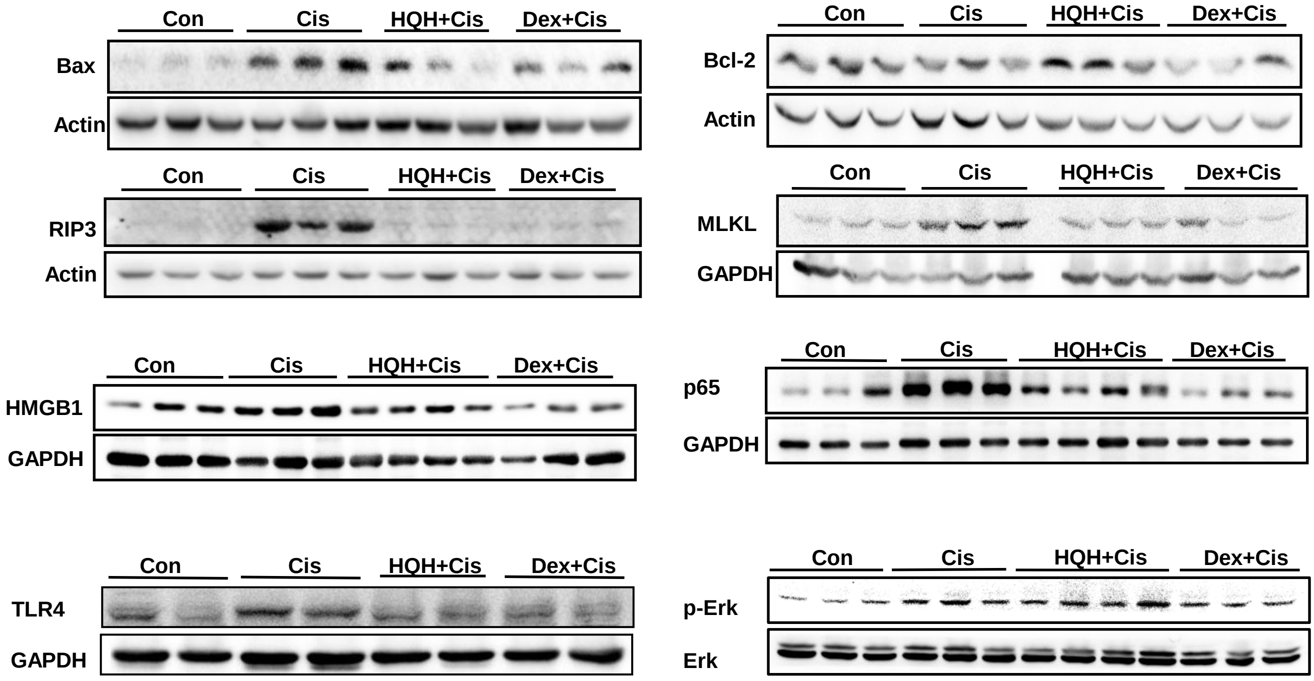

Supplement：The relevant target protein and internal control protein shown in the immunoblot are the same gel, the same exposure.

Pretreatment of Huaiqihuang extractum protects against cisplatin-induced nephrotoxicity

Yujiao Guo[1,2], Meng Wang [1], Jingyi Mou[3], Zhi Zhao[1], Juan Yang[1], Fengming Zhu [1],Guangchang Pei[1], Han Zhu[1], Yuxi Wang[1], Gang Xu[1], Rui Zeng[1,*], Ying Yao[1,*]
[1]Department of Nephrology, Tongji Hospital, Tongji Medical College, Huazhong University of Science and Technology
[2]Department of Radiology, Tongji Hospital, Tongji Medical College, Huazhong University of Science and Technology
[3]Department of Pediatric, Tongji Hospital, Tongji Medical College, Huazhong University of Science and Technology
*They contributed equally to corresponding author, 1095 Jiefang Ave, Wuhan, Hubei, China (postcode, 430030). (YY) E-mail:yaoyingkk@126.com; (RZ)E-mail:zengrui@tjh.tjmu.edu.cn

Original scans

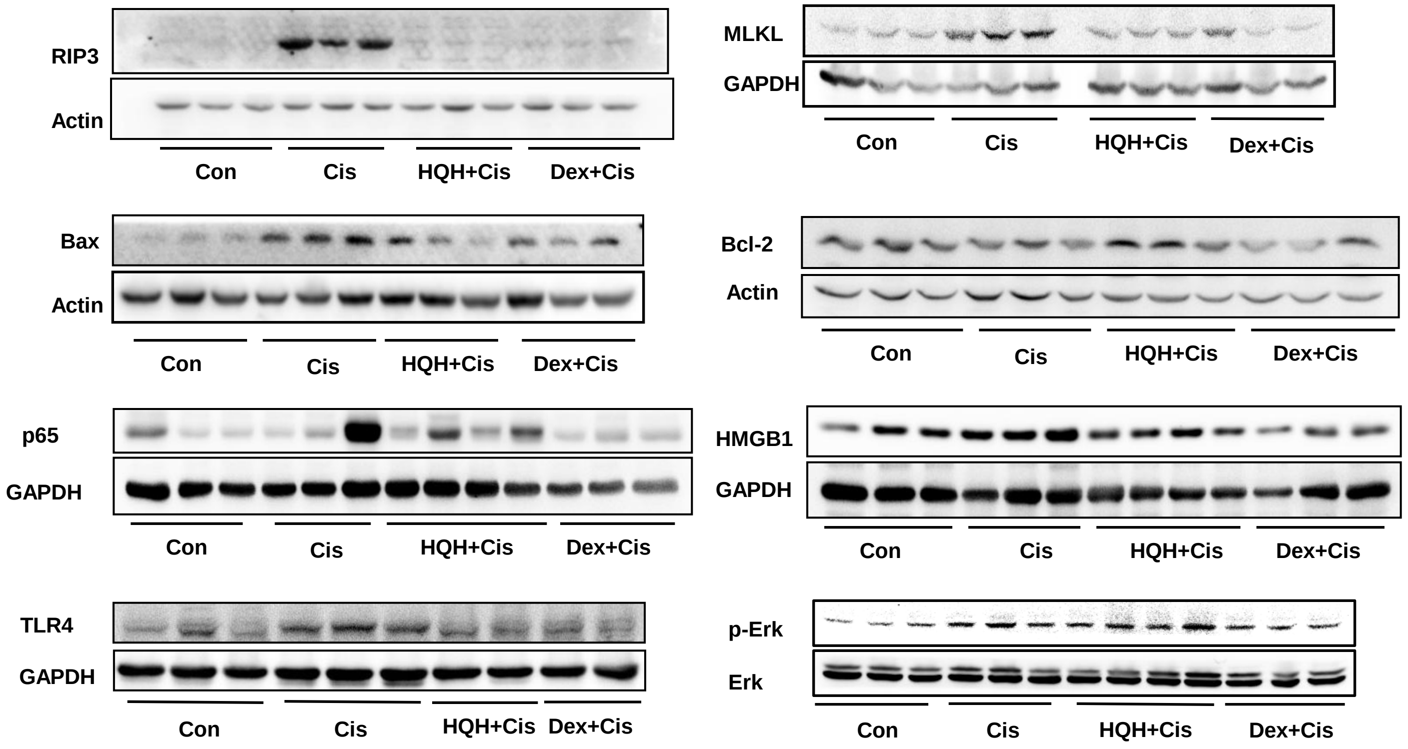

Supplement: Supplementary file 1 — Supplementary Information [file 41598_2018_25610_MOESM1_ESM.pdf]
